# Supplementary material for: IMBAS-MS Discovers Organ-Specific HLA Peptide Patterns in Plasma
Source: Mol Cell Proteomics. 2023 Dec 1;23(1):100689. doi: 10.1016/j.mcpro.2023.100689 (PMC10765297; doi:10.1016/j.mcpro.2023.100689)
Supplement: Supplemental data [file mmc1.pdf]

Supplementary Table 1: Donor identifier with associated HLA-type including Figure assignments.

Donor	HLA-type	Figure 1	Figure 2	Figure 3	Figure 5
1	HLA-A24:02,HLA-A26:01,HLA-B40:01,HLA-B39:01,HLA-C03:04,HLA-C12:03	x	x	x (Donor 1)	x (Donor 1)
2	HLA-A24:02, HLA-A31:01, HLA-B27:07, HLA-B50:01, HLA-C06:02, HLA-C15:02	-	-	x (Donor 2)	x (Donor 2)
3	HLA-A02:01, HLA-A32:01, HLA-B15:01, HLA-B35:01, HLA-C03:04, HLA-C04:01	-	-	x (Donor 3)	x (Donor 3)
4	HLA-A02:01,HLA-A32:01,HLA-B08:01,HLA-B35:03,HLA-C04:01,HLA-C07:01	-	-	x (Donor 5)	x (Donor 4);timeline*
5	HLA-A02:06, HLA-A11:01, HLA-B15:13, HLA-B40:01, HLA-C08:01, HLA-C15:02	-	-	-	x (Donor 5)
6	HLA-A01:01, HLA-A25:01, HLA-B07:02, HLA-B55:01, HLA-C03:03, HLA-C07:02	-	-	-	x (Donor 6)
7	HLA-A24:02, HLA-A30:01, HLA-B07:43, HLA-B07:236, HLA-C07:02, HLA-C08:02	-	-	x (Donor 6)	x (Donor 7)
8	HLA-A01:01, HLA-A24:03,HLA-B38:20, HLA-B57:01, HLA-C06:02, HLA-C12:03	-	-	x (Donor 4)	x (Donor 8)

* 11 month sampling point post mild COVID-19 infection (confirmed by antigen test) was taken 3 days after first negative antigen test.

A)

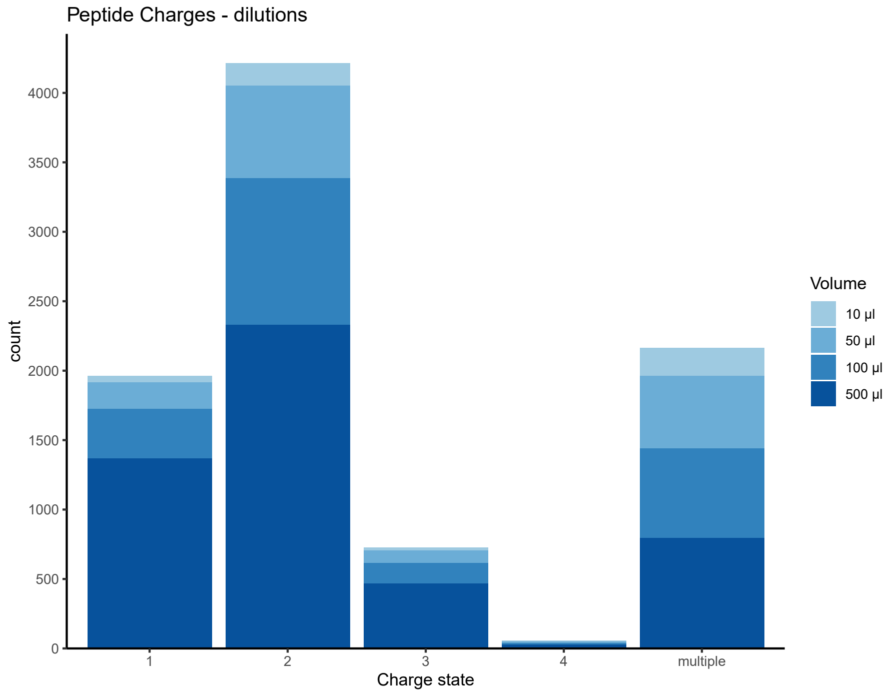

Peptide Charges - dilutions
count
0
500
1000
1500
2000
2500
3000
3500
4000
1
2
3
4
multiple
Charge state
Volume
10 µl
50 µl
100 µl
500 µl

B)

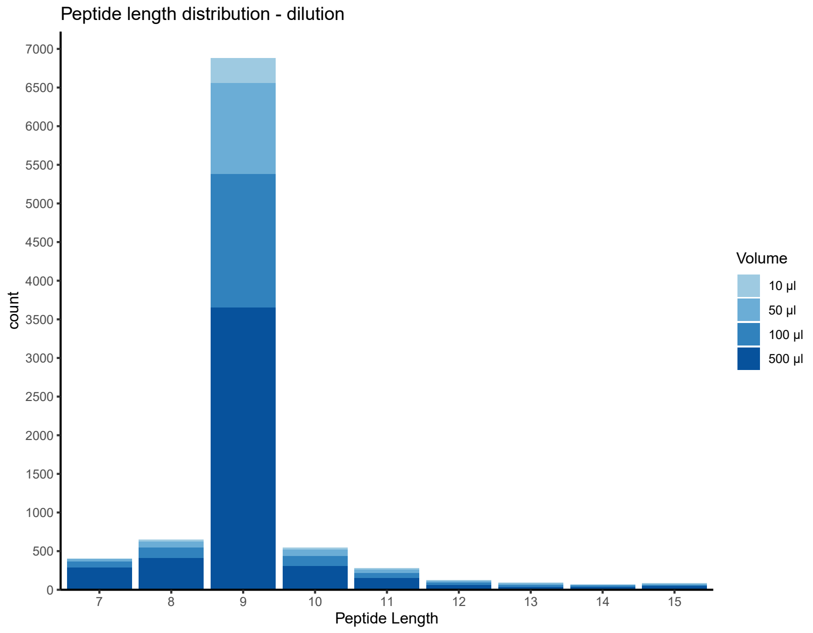

Peptide length distribution - dilution
count
0
500
1000
1500
2000
2500
3000
3500
4000
4500
5000
5500
6000
6500
7000
7
8
9
10
11
12
13
14
15
Peptide Length
Volume
10 µl
50 µl
100 µl
500 µl

Supplementary Figure 1: Length and charge properties of IMBAS-MS DDA based identifications. (A) Stacked bar plot of charge states of all identified precursors identified in the dilution series experiment (Figure 1C). **(B)** Stacked bar plot of length distribution of identified HLA class I peptides of all samples in (Figure 1C).

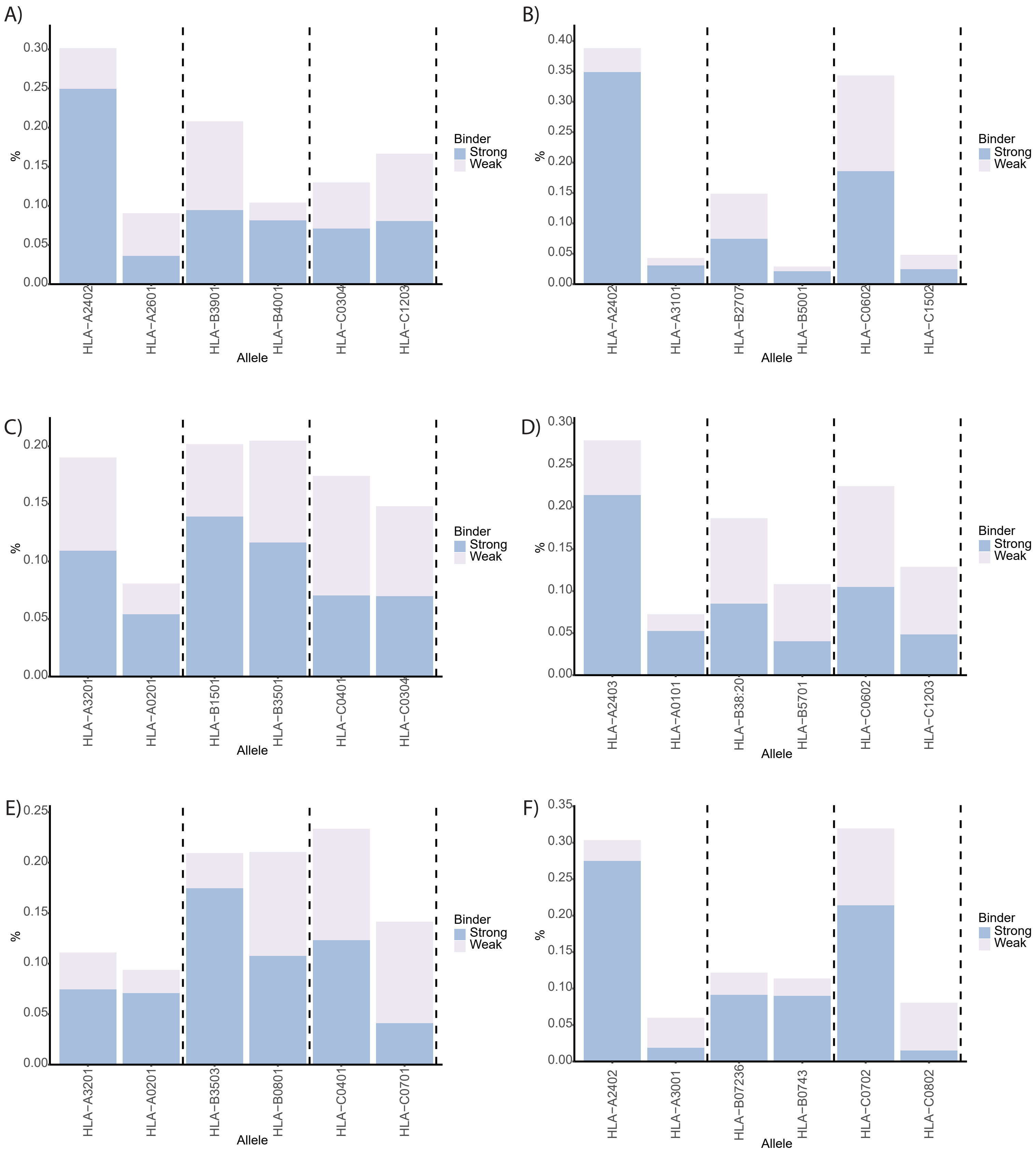
A)
%
Allele
HLA−A2402
HLA−A2601
HLA−B3901
HLA−B4001
HLA−C0304
HLA−C1203
Binder
Strong
Weak
B)
%
Allele
HLA−A2402
HLA−A3101
HLA−B2707
HLA−B5001
HLA−C0602
HLA−C1502
Binder
Strong
Weak
C)
%
Allele
HLA−A3201
HLA−A0201
HLA−B1501
HLA−B3501
HLA−C0401
HLA−C0304
Binder
Strong
Weak
D)
%
Allele
HLA−A2403
HLA−A0101
HLA−B38:20
HLA−B5701
HLA−C0602
HLA−C1203
Binder
Strong
Weak
E)
%
Allele
HLA−A3201
HLA−A0201
HLA−B3503
HLA−B0801
HLA−C0401
HLA−C0701
Binder
Strong
Weak
F)
%
Allele
HLA−A2402
HLA−A3001
HLA−B07236
HLA−B0743
HLA−C0702
HLA−C0802
Binder
Strong
Weak

Supplementary Figure 2: Relative distribution of HLA peptides within every donor. (A) – (F) Stacked bar plot of the relative amount of strong and weak binders per HLA type in relation to the total amount of HLA peptides identified per person for donor#1 to donor#6

A)

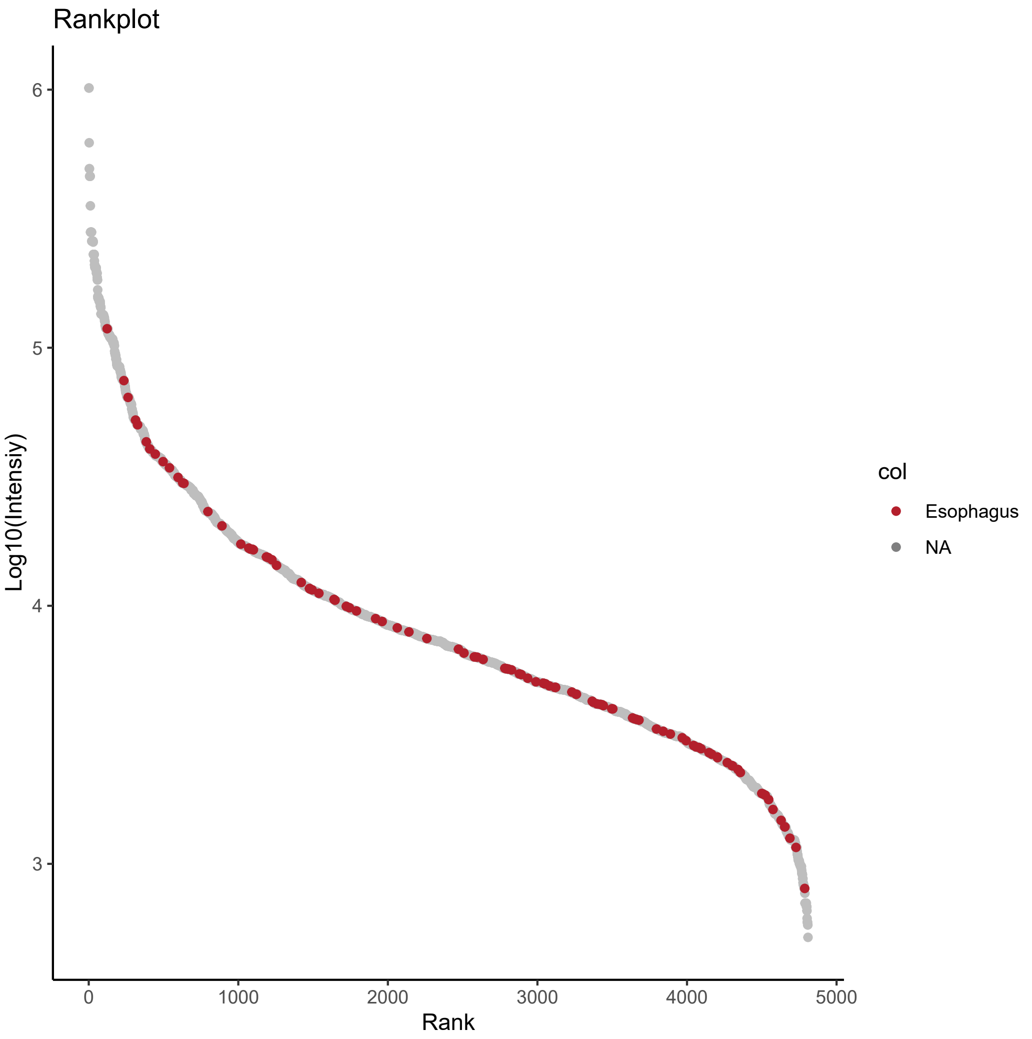

Rankplot
Log10(Intensiy)
6
5
4
3
0
1000
2000
3000
4000
5000
Rank
col
Esophagus
NA

B)

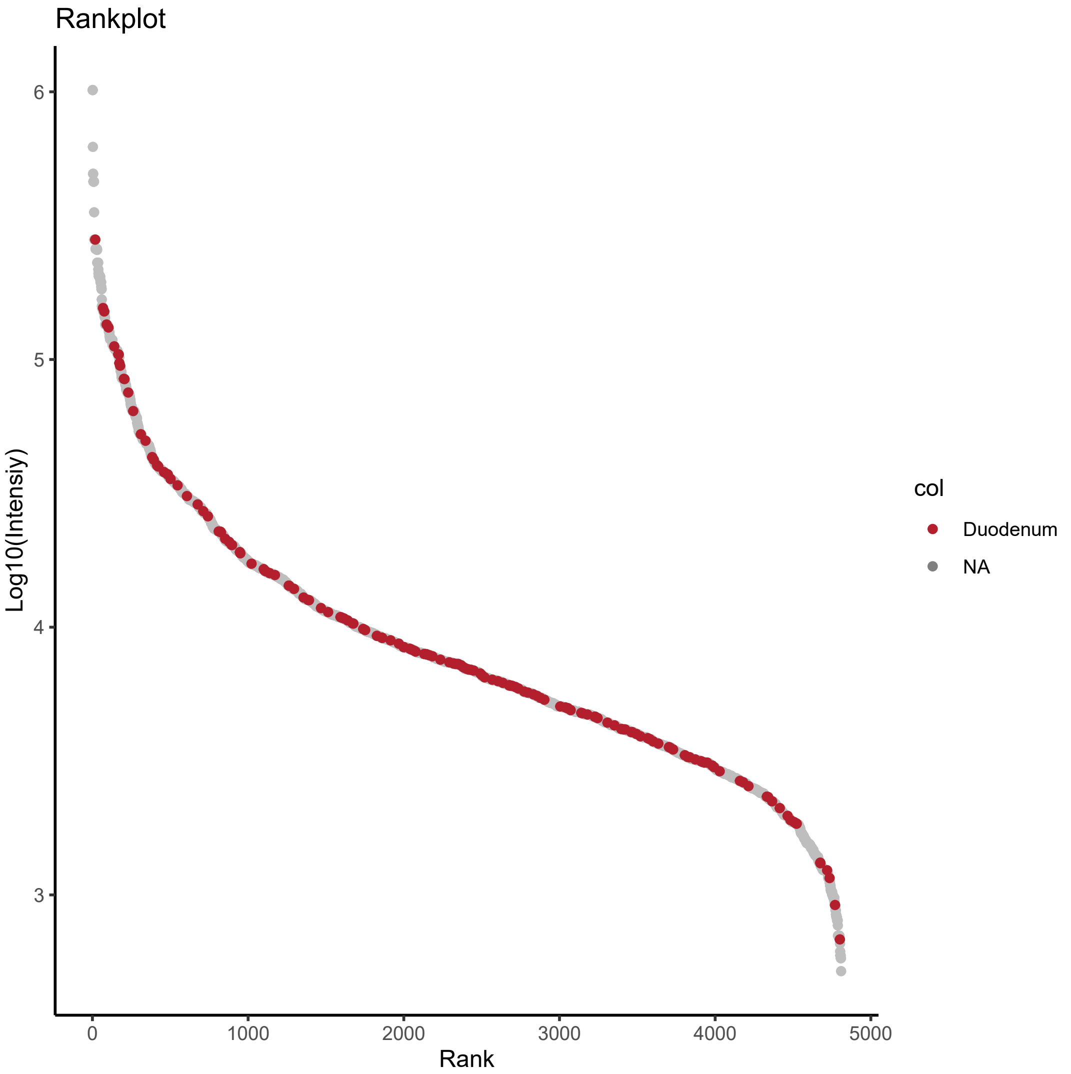

Rankplot
Log10(Intensiy)
6
5
4
3
0
1000
2000
3000
4000
5000
Rank
col
Duodenum
NA

C)

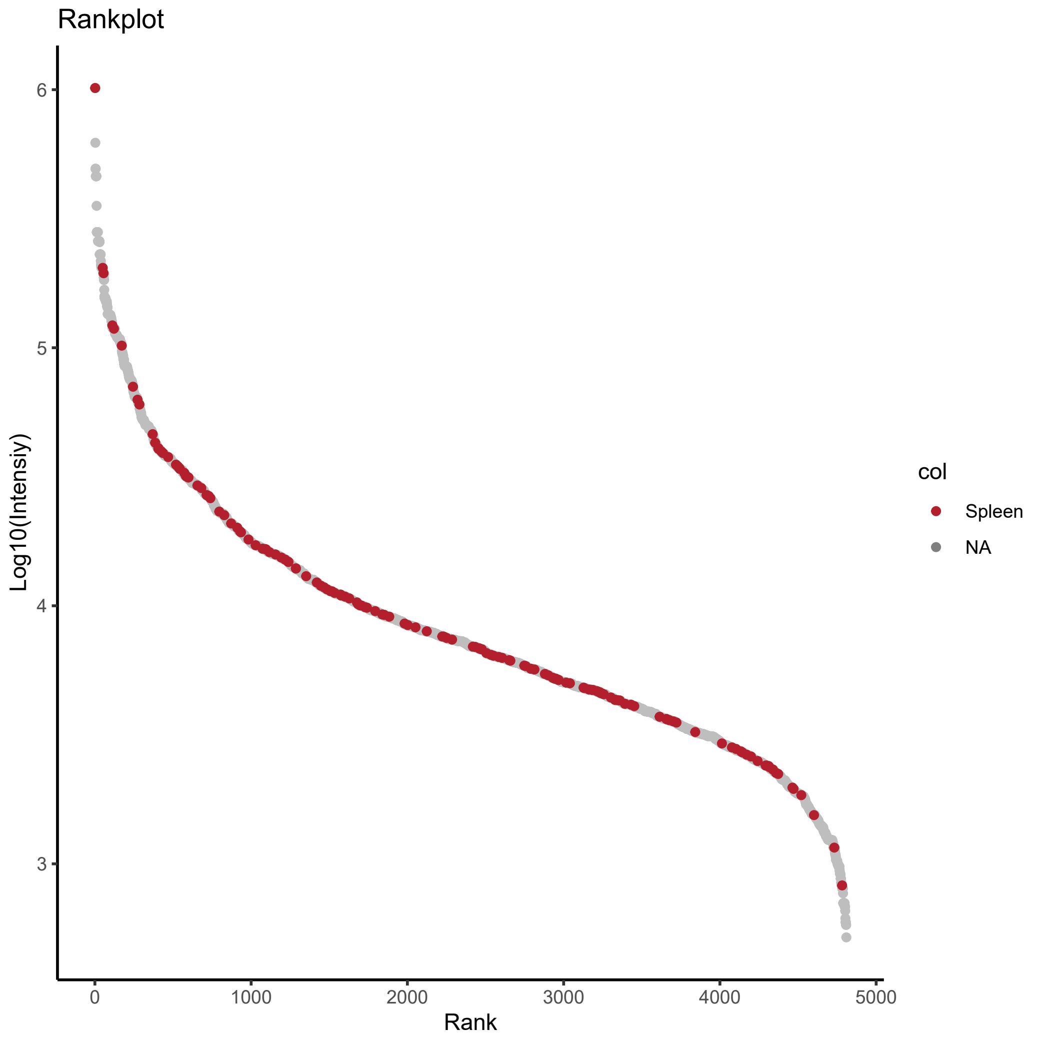

Rankplot
Log10(Intensiy)
6
5
4
3
0
1000
2000
3000
4000
5000
Rank
col
Spleen
NA

D)

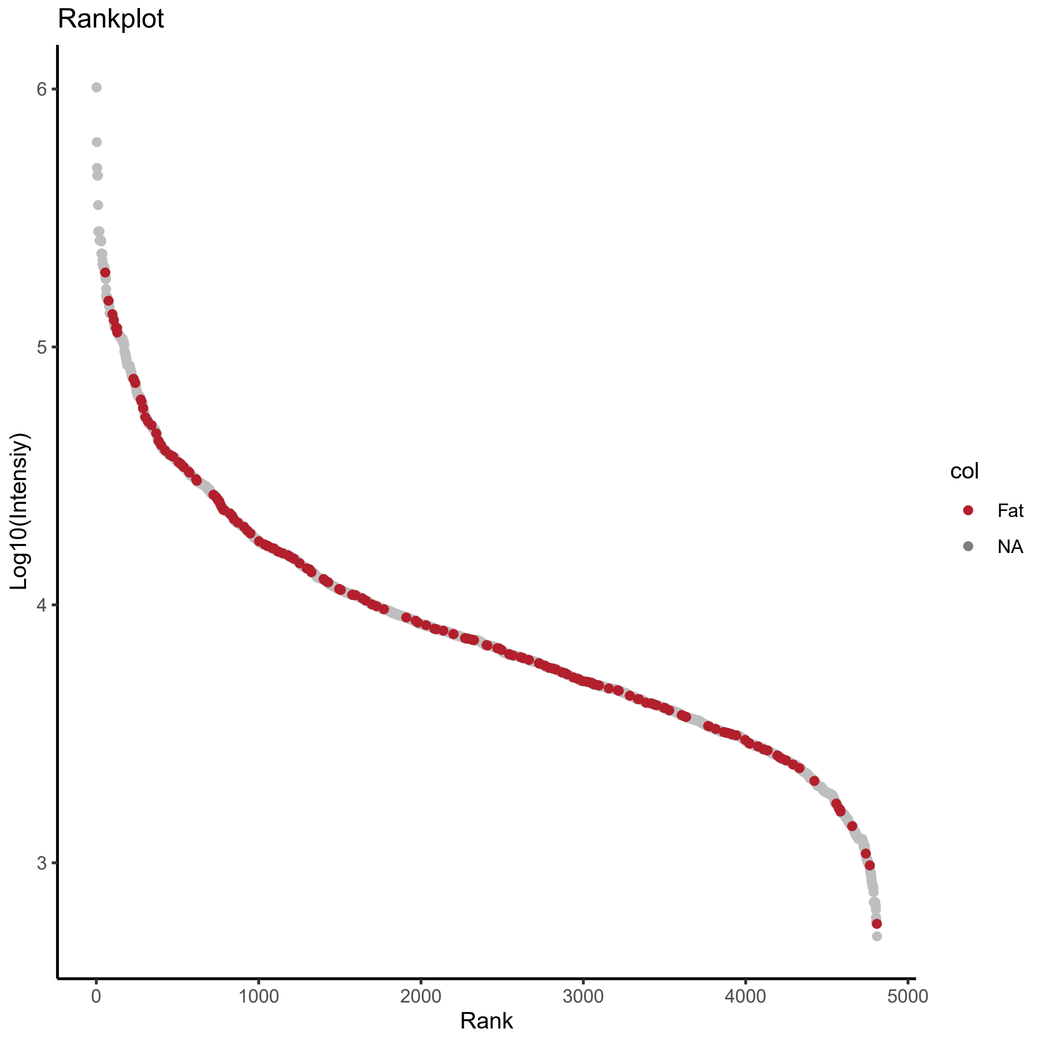

Rankplot
Log10(Intensiy)
6
5
4
3
0
1000
2000
3000
4000
5000
Rank
col
Fat
NA

E)

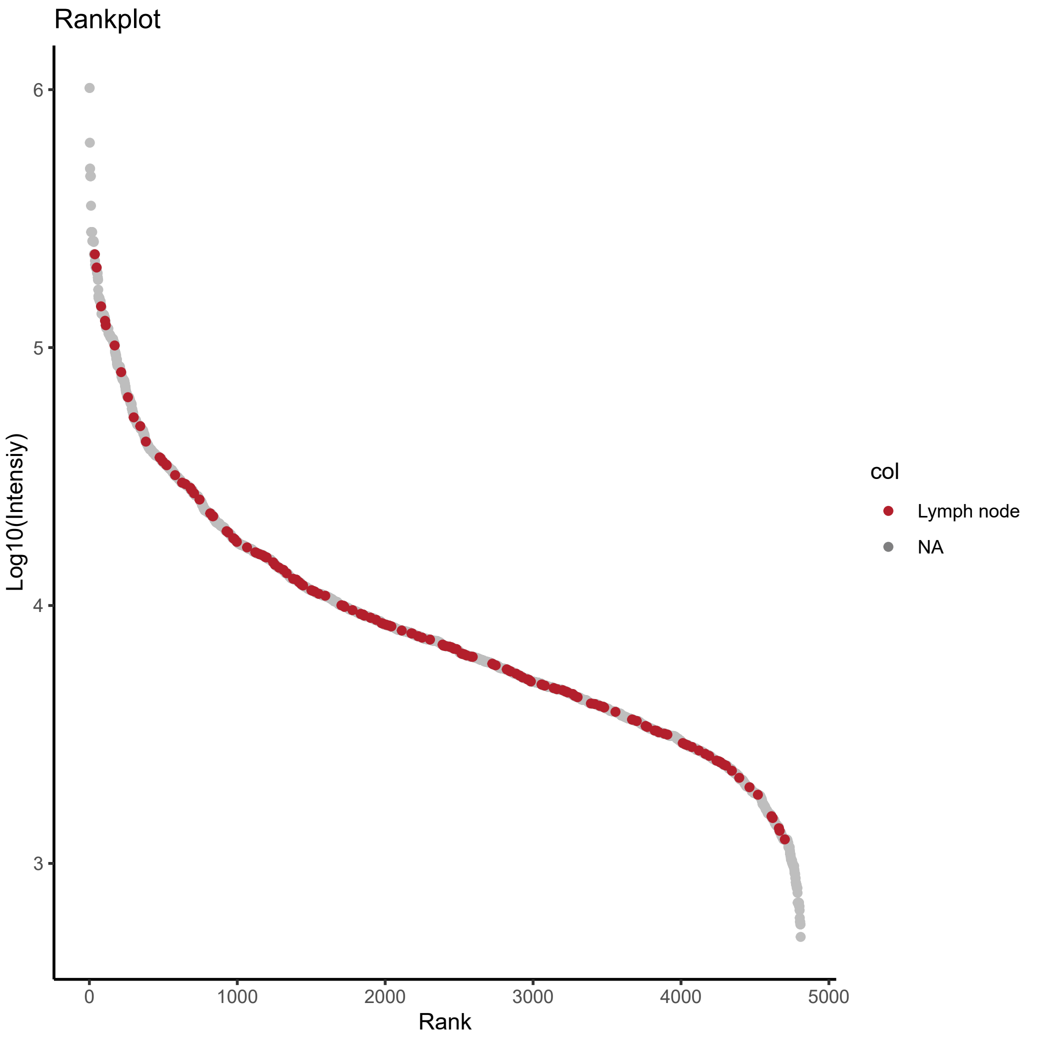
Rankplot
Log10(Intensiy)
6
5
4
3
0
1000
2000
3000
4000
5000
Rank
col
Lymph node
NA

F)

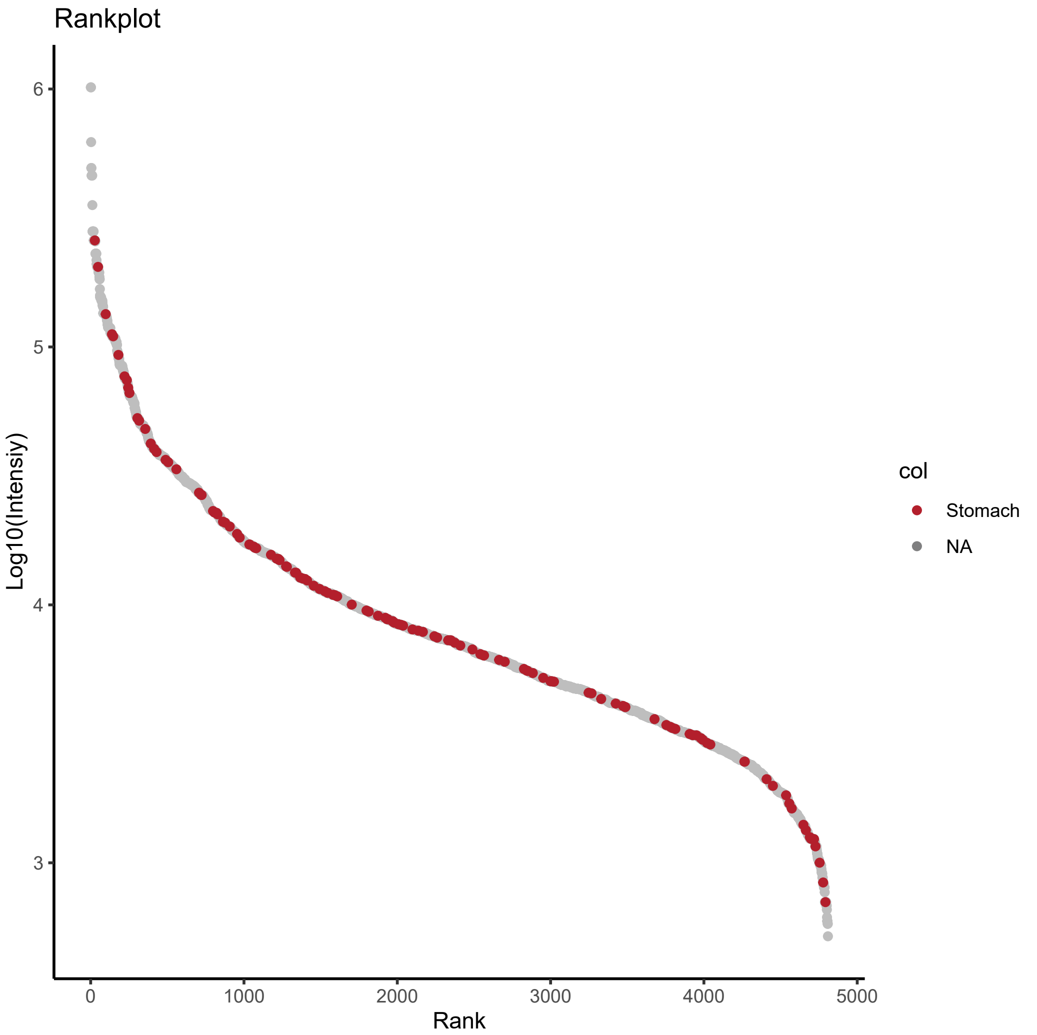
Rankplot
Log10(Intensiy)
6
5
4
3
0
1000
2000
3000
4000
5000
Rank
col
Stomach
NA

I)

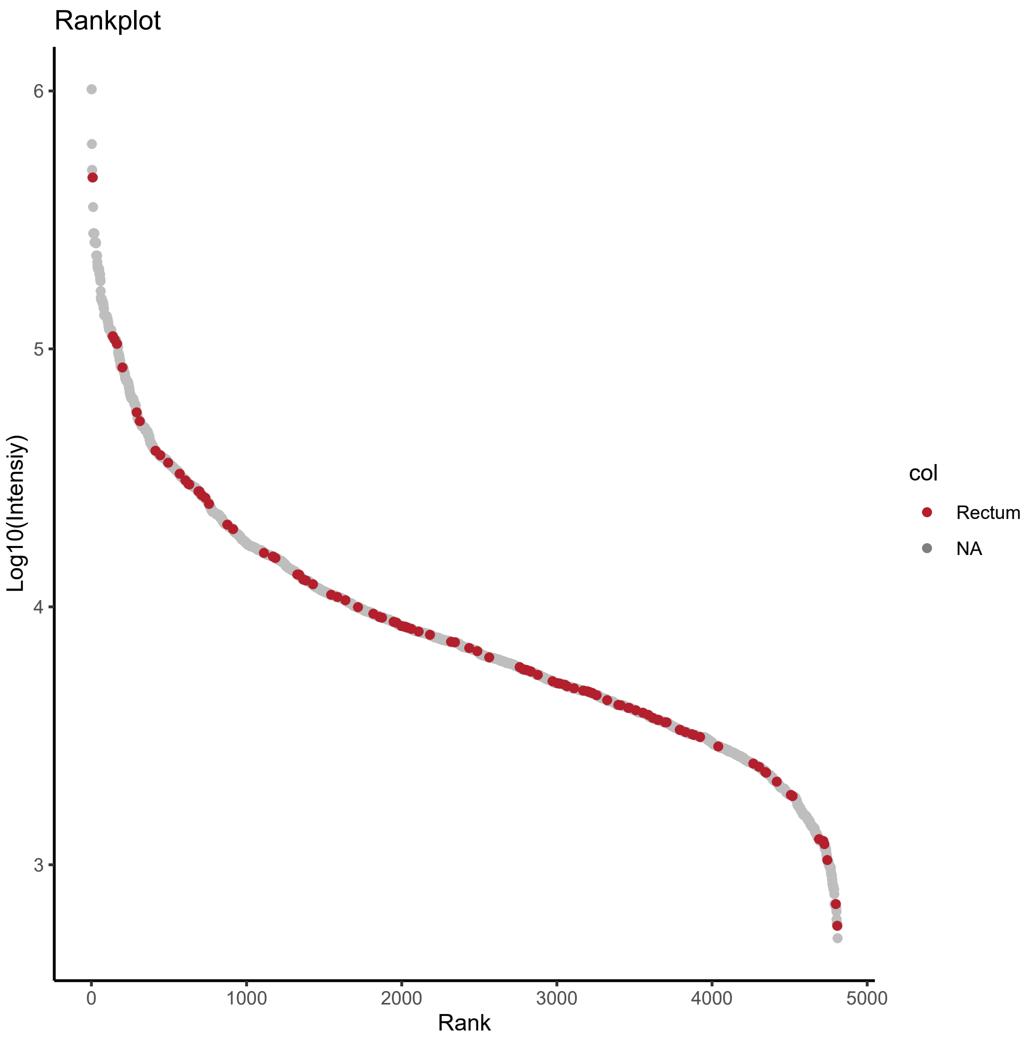
Rankplot
Log10(Intensiy)
6
5
4
3
0
1000
2000
3000
4000
5000
Rank
col
Rectum
NA

J)

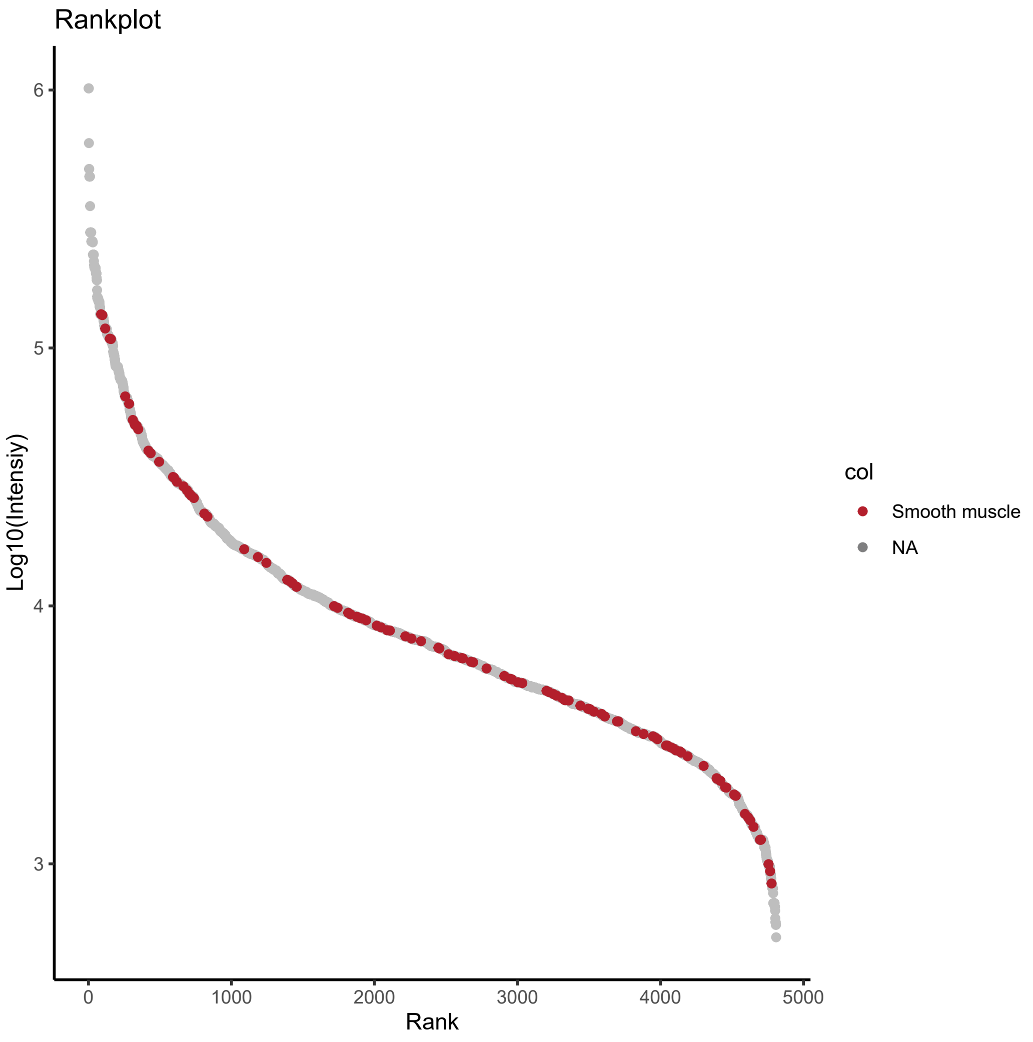
Rankplot
Log10(Intensiy)
6
5
4
3
0
1000
2000
3000
4000
5000
Rank
col
Smooth muscle
NA

K)

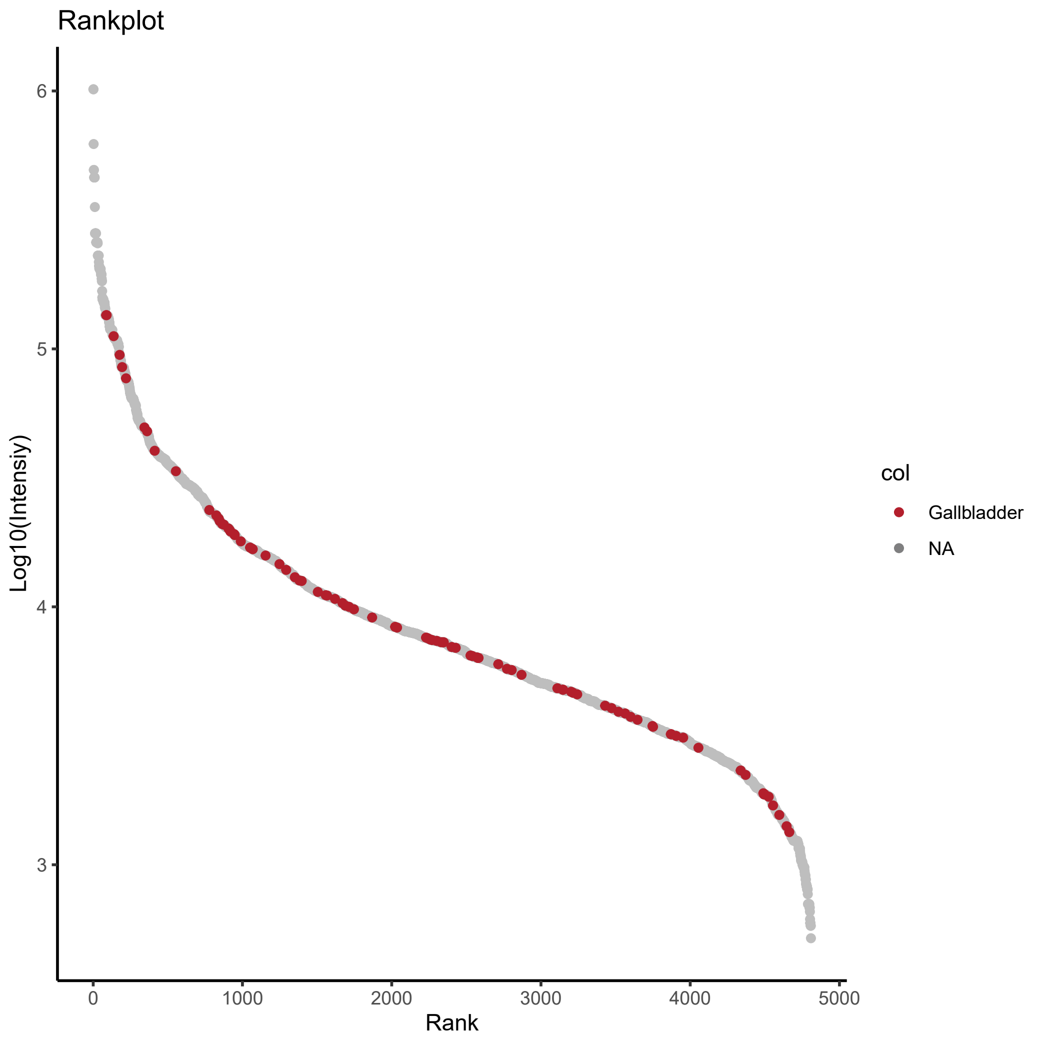
Rankplot
6
5
4
3
Log10(Intensiy)
0
1000
2000
3000
4000
5000
Rank
col
Gallbladder
NA

L)

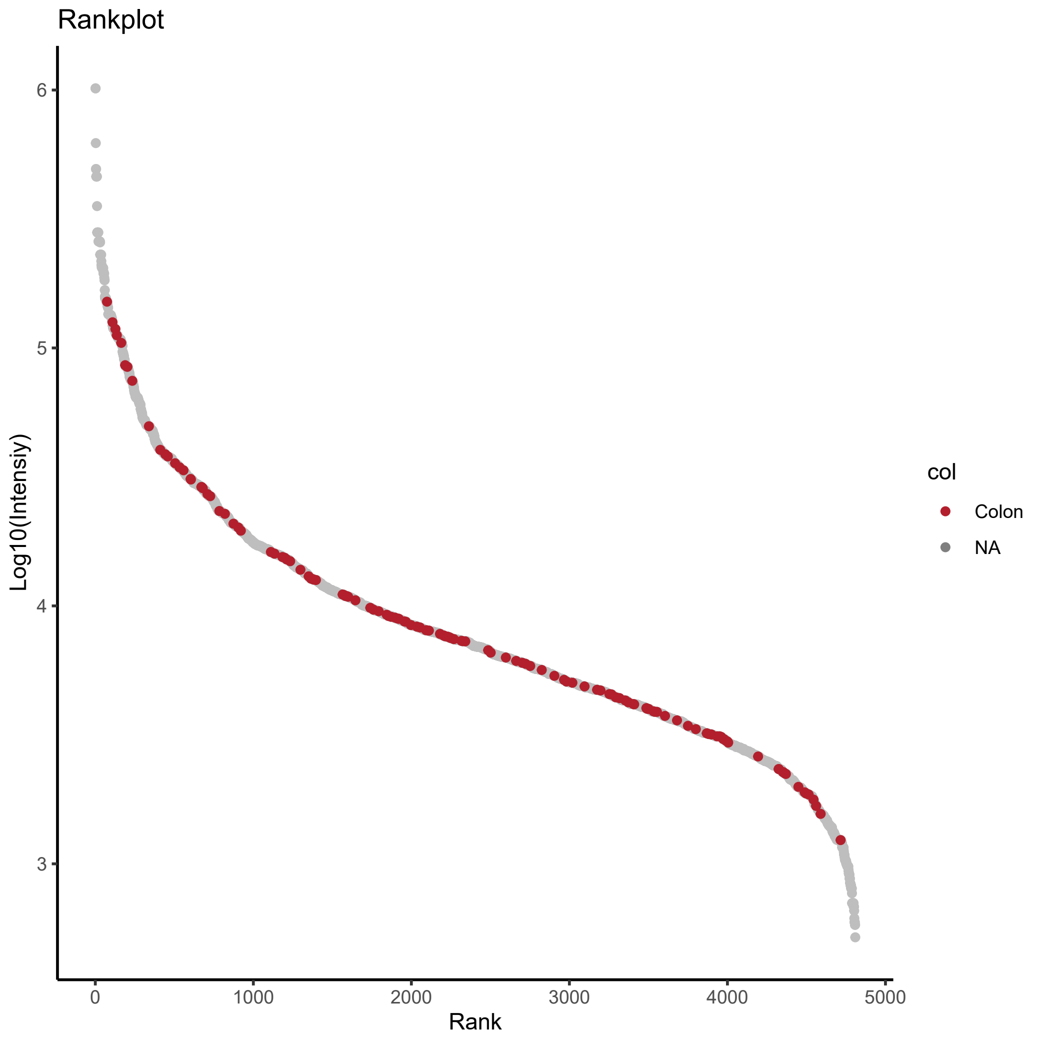
Rankplot
6
5
4
3
Log10(Intensiy)
0
1000
2000
3000
4000
5000
Rank
col
Colon
NA

M)

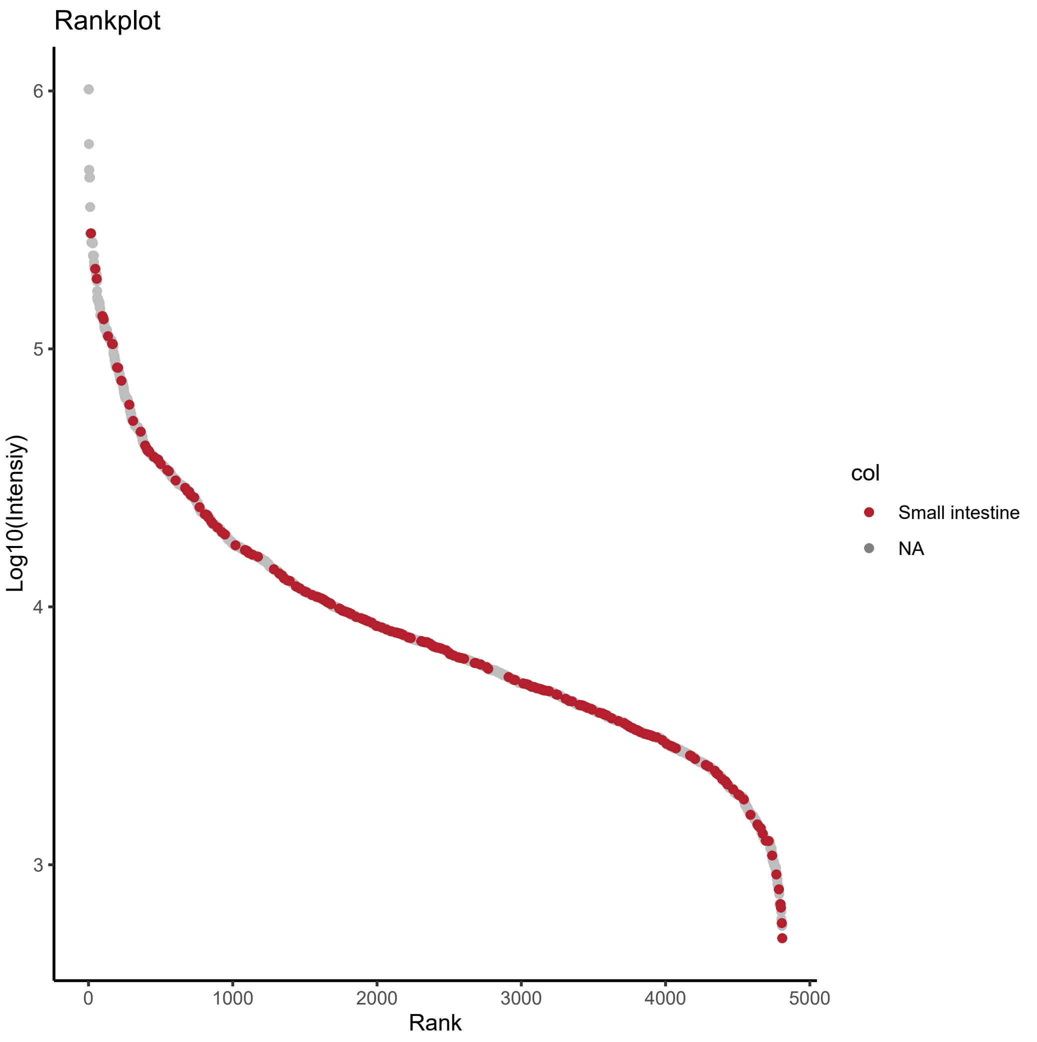
Rankplot
Log10(Intensiy)
6
5
4
3
0
1000
2000
3000
4000
5000
Rank
col
Small intestine
NA

N)

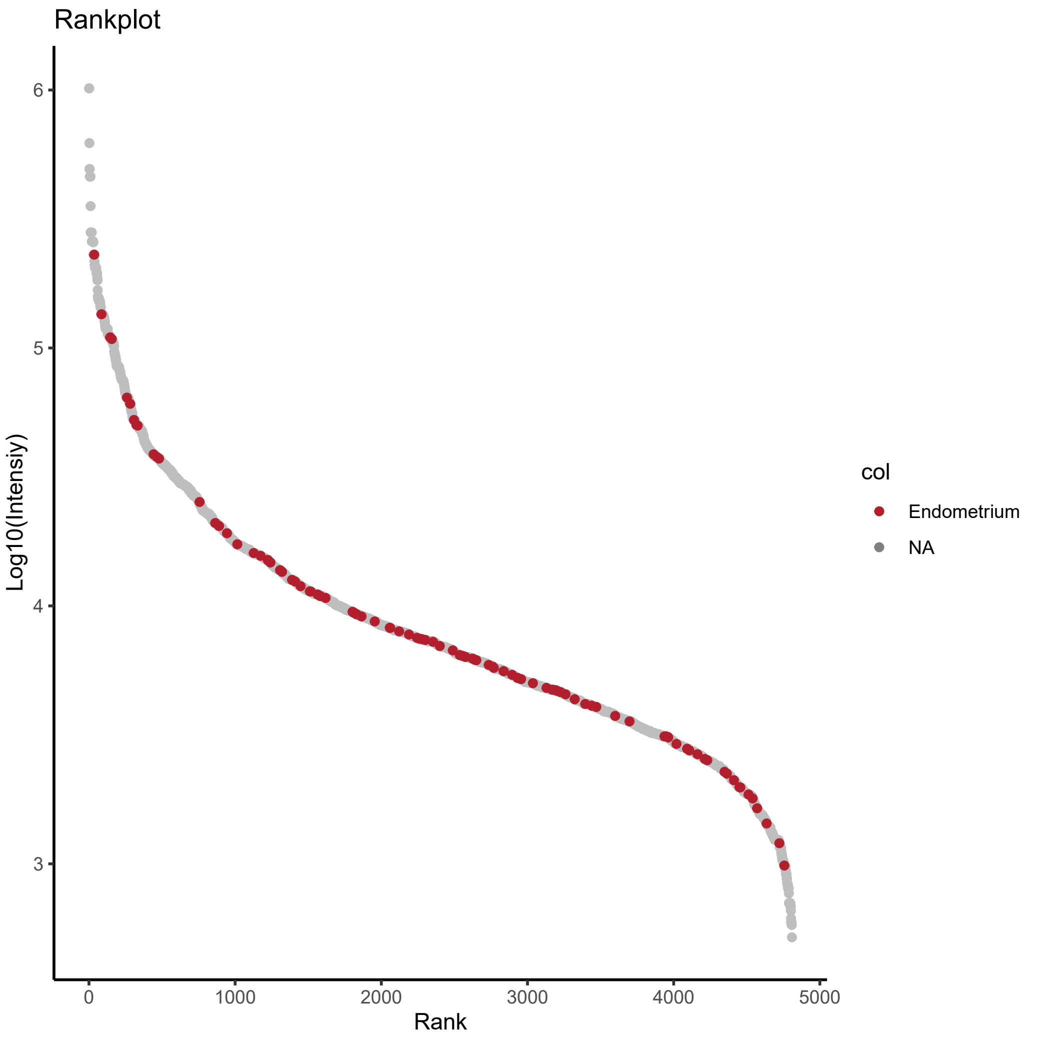
Rankplot
Log10(Intensiy)
6
5
4
3
0
1000
2000
3000
4000
5000
Rank
col
Endometrium
NA

G)

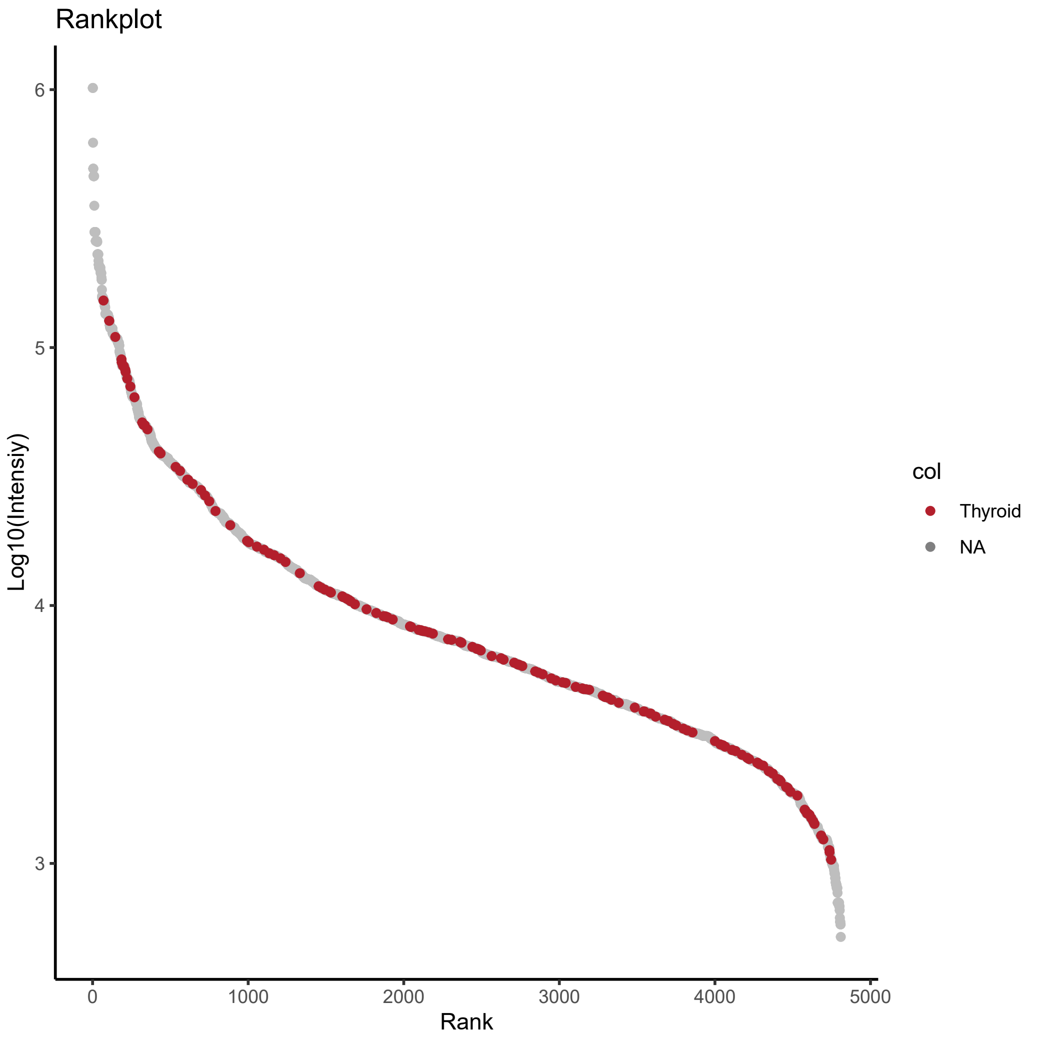
Rankplot
Log10(Intensiy)
6
5
4
3
0
1000
2000
3000
4000
5000
Rank
col
Thyroid
NA

H)

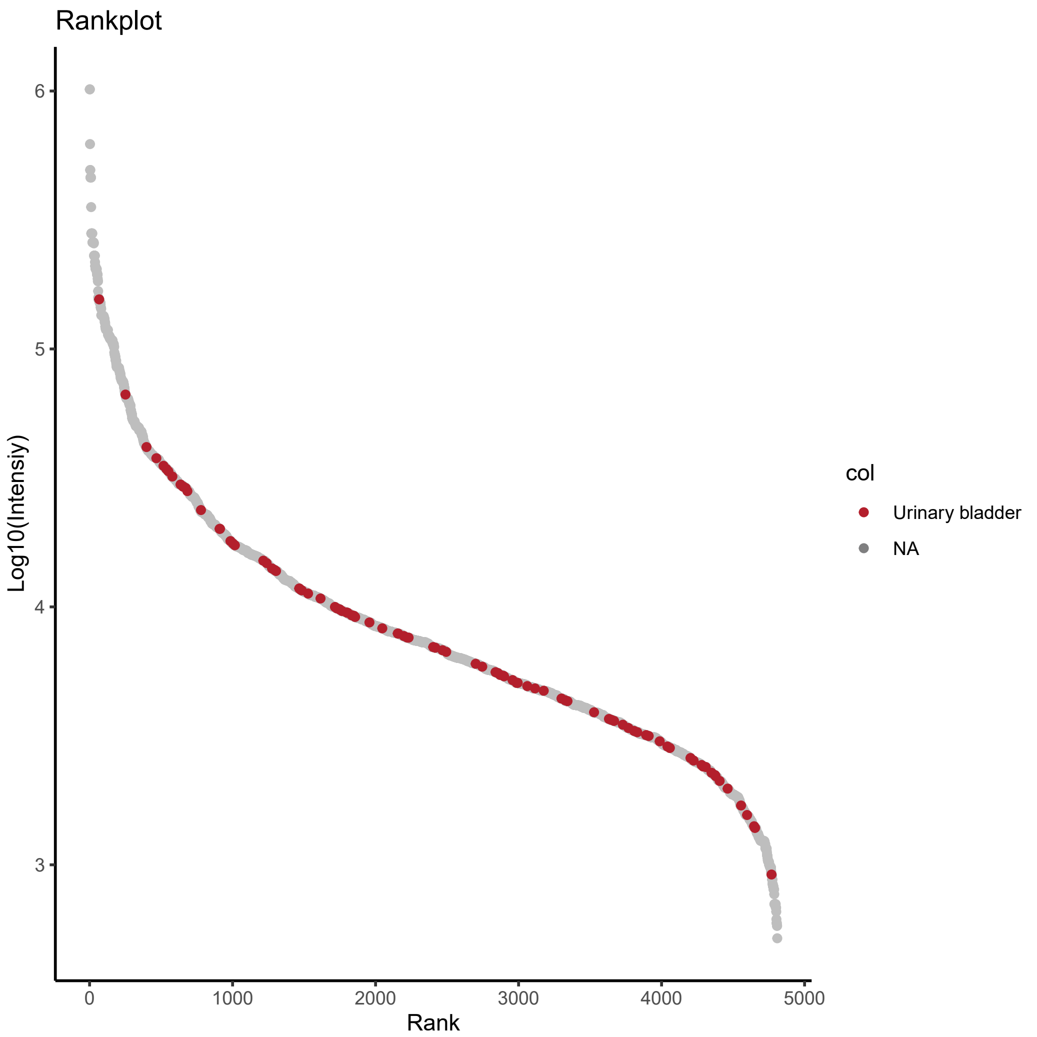
Rankplot
Log10(Intensiy)
6
5
4
3
0
1000
2000
3000
4000
5000
Rank
col
Urinary bladder
NA

O)

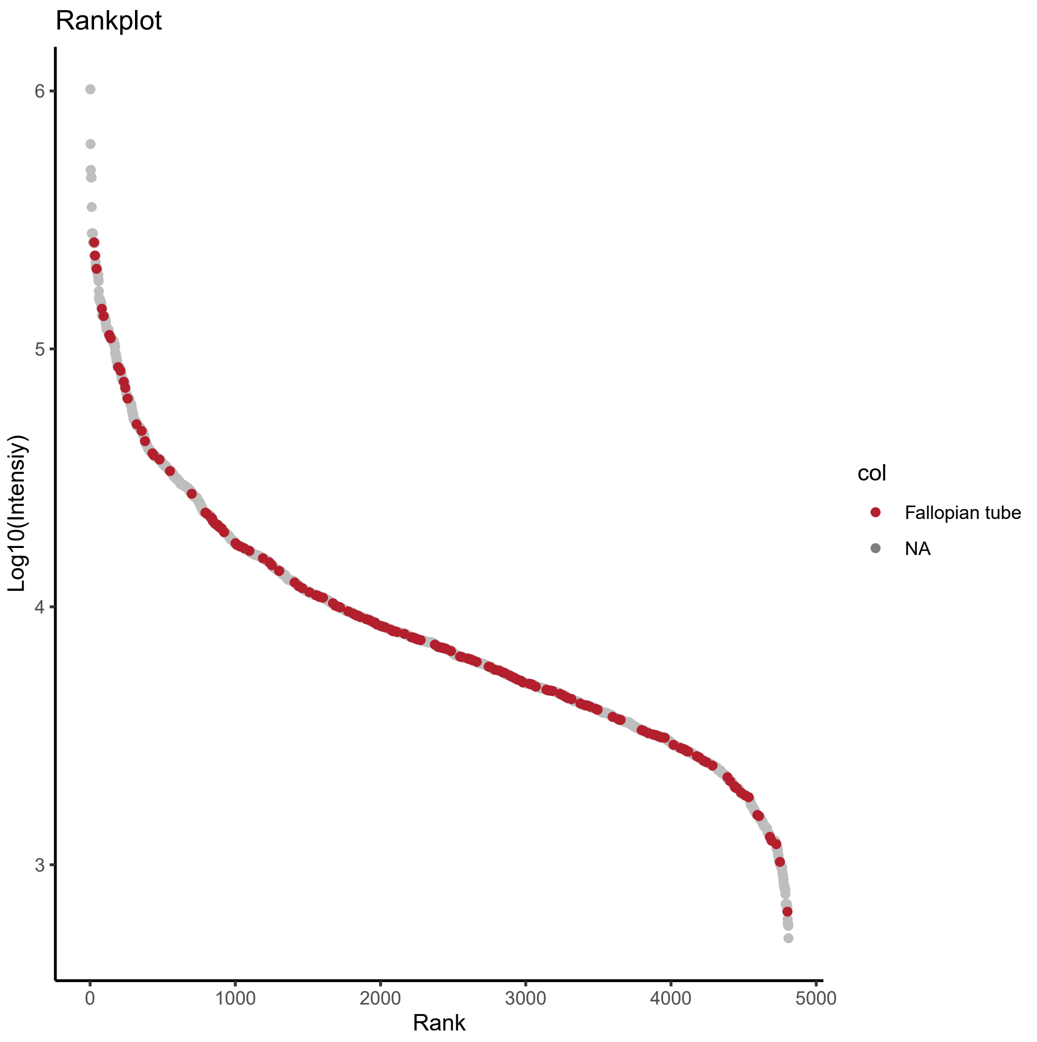
Rankplot
Log10(Intensiy)
6
5
4
3
0
1000
2000
3000
4000
5000
Rank
col
Fallopian tube
NA

P)

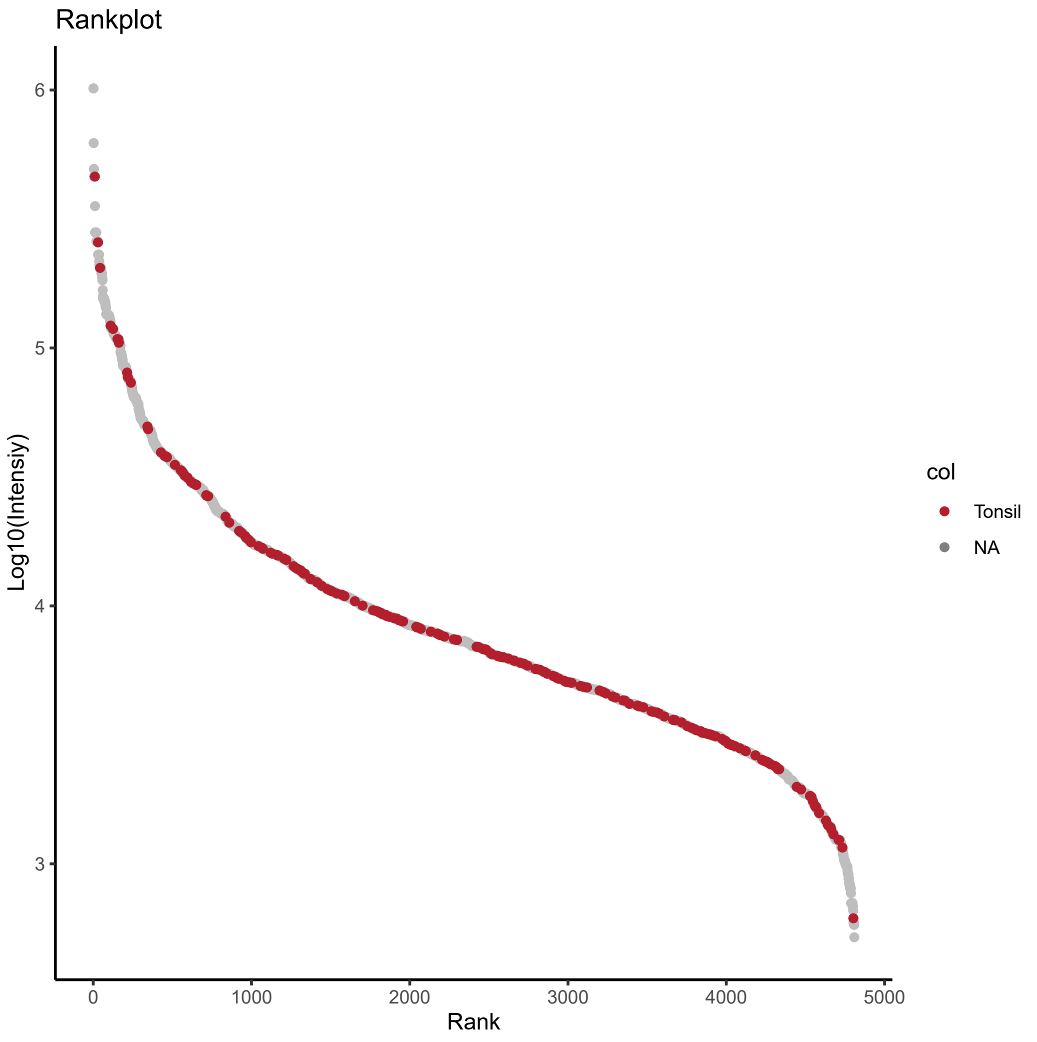
Rankplot
Log10(Intensiy)
6
5
4
3
0
1000
2000
3000
4000
5000
Rank
col
Tonsil
NA

Q)

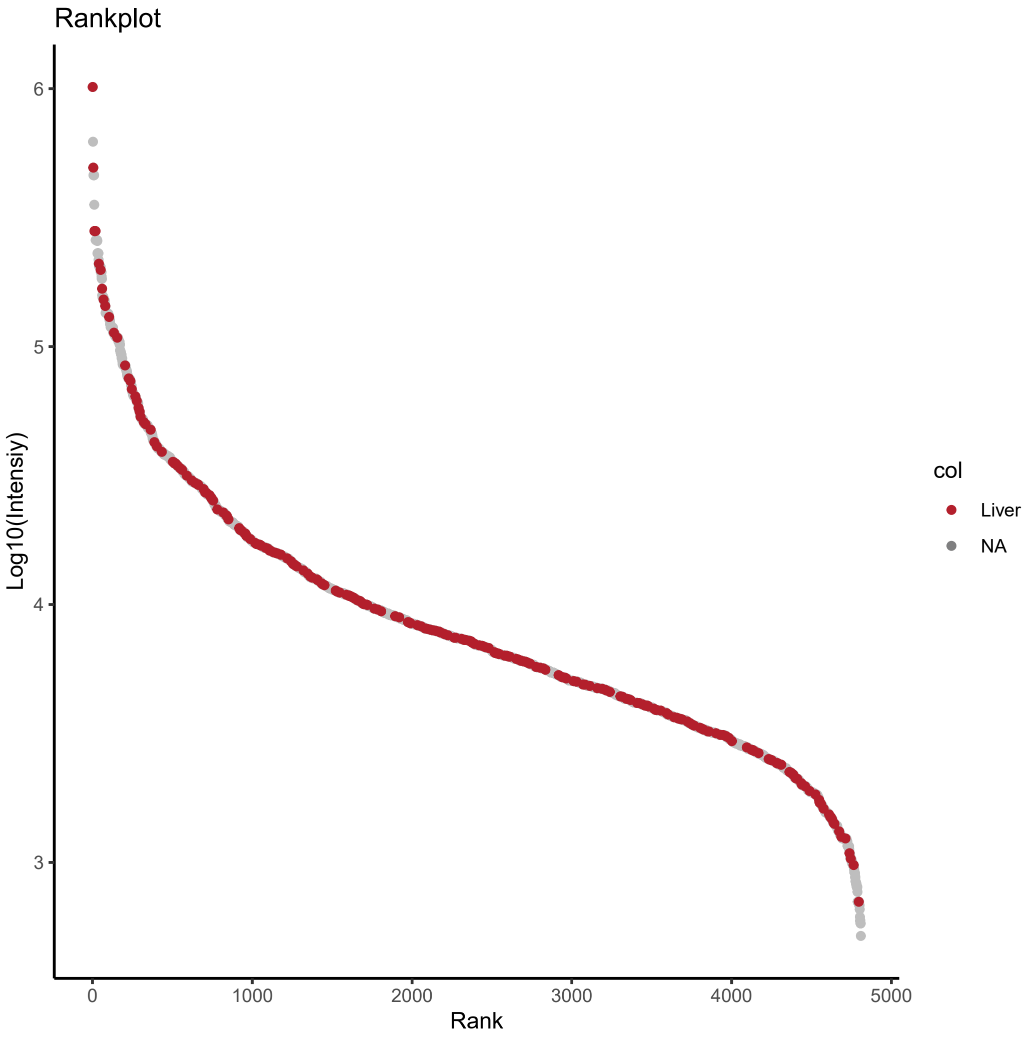
Rankplot
6
5
4
3
Log10(Intensiy)
0
1000
2000
3000
4000
5000
Rank
col
Liver
NA

R)

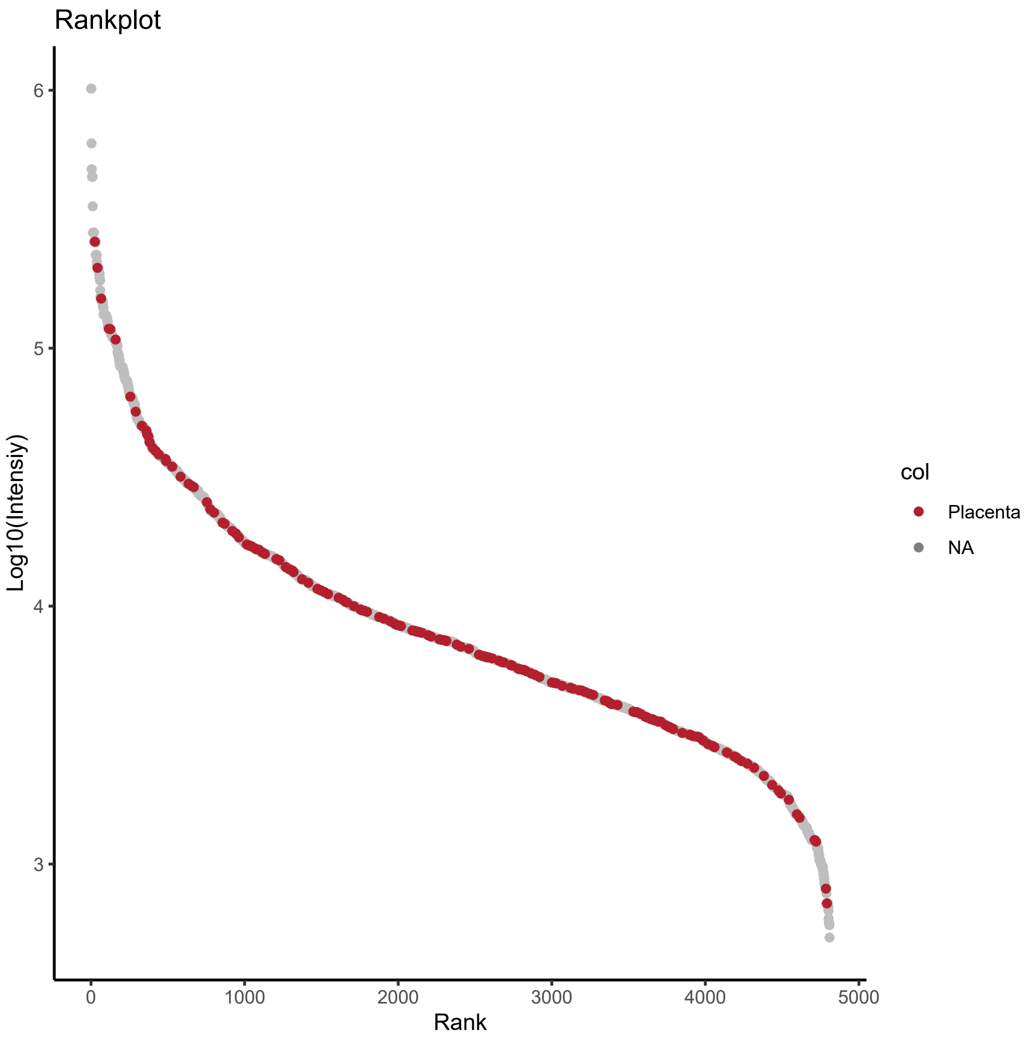
Rankplot
6
5
4
3
Log10(Intensiy)
0
1000
2000
3000
4000
5000
Rank
col
Placenta
NA

S)

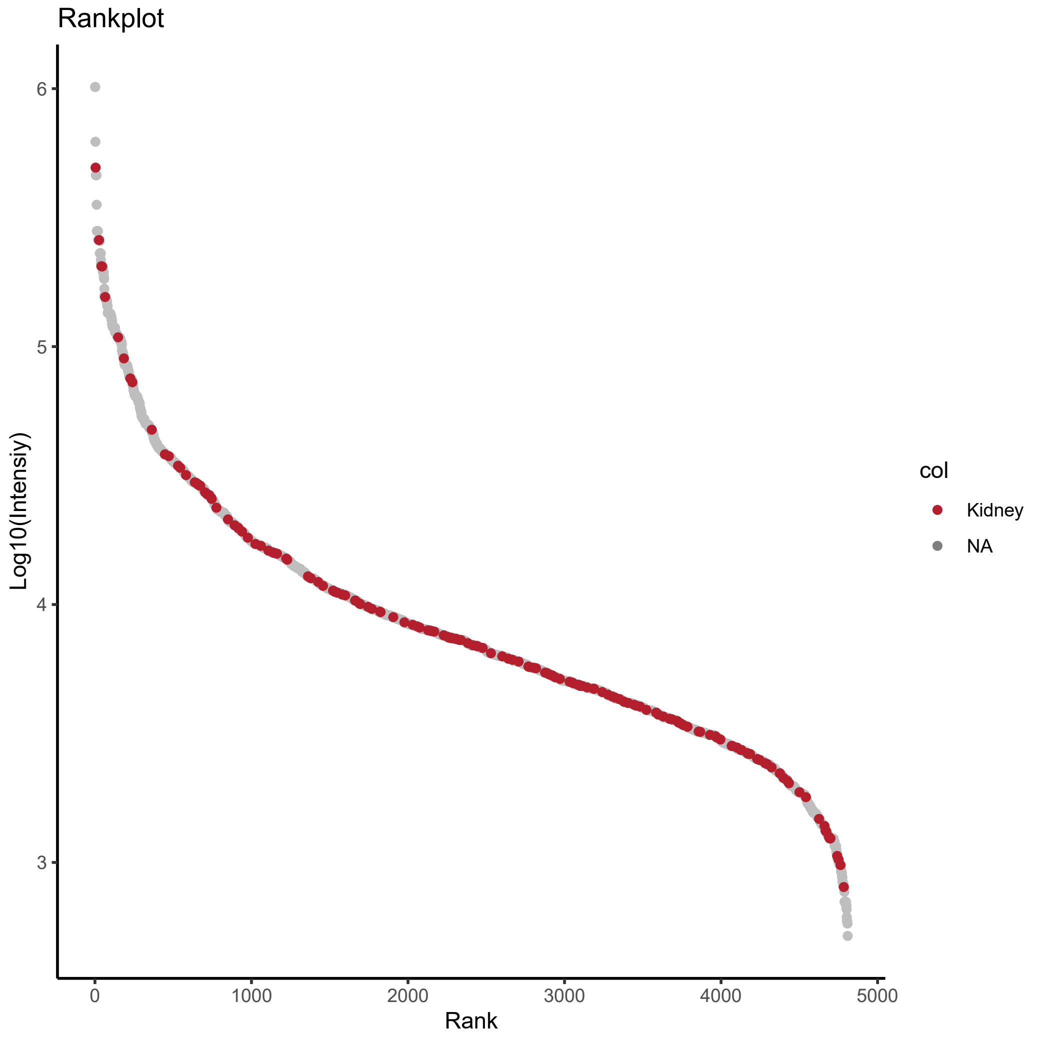
Rankplot
6
5
4
3
Log10(Intensiy)
0
1000
2000
3000
4000
5000
Rank
col
Kidney
NA

T)

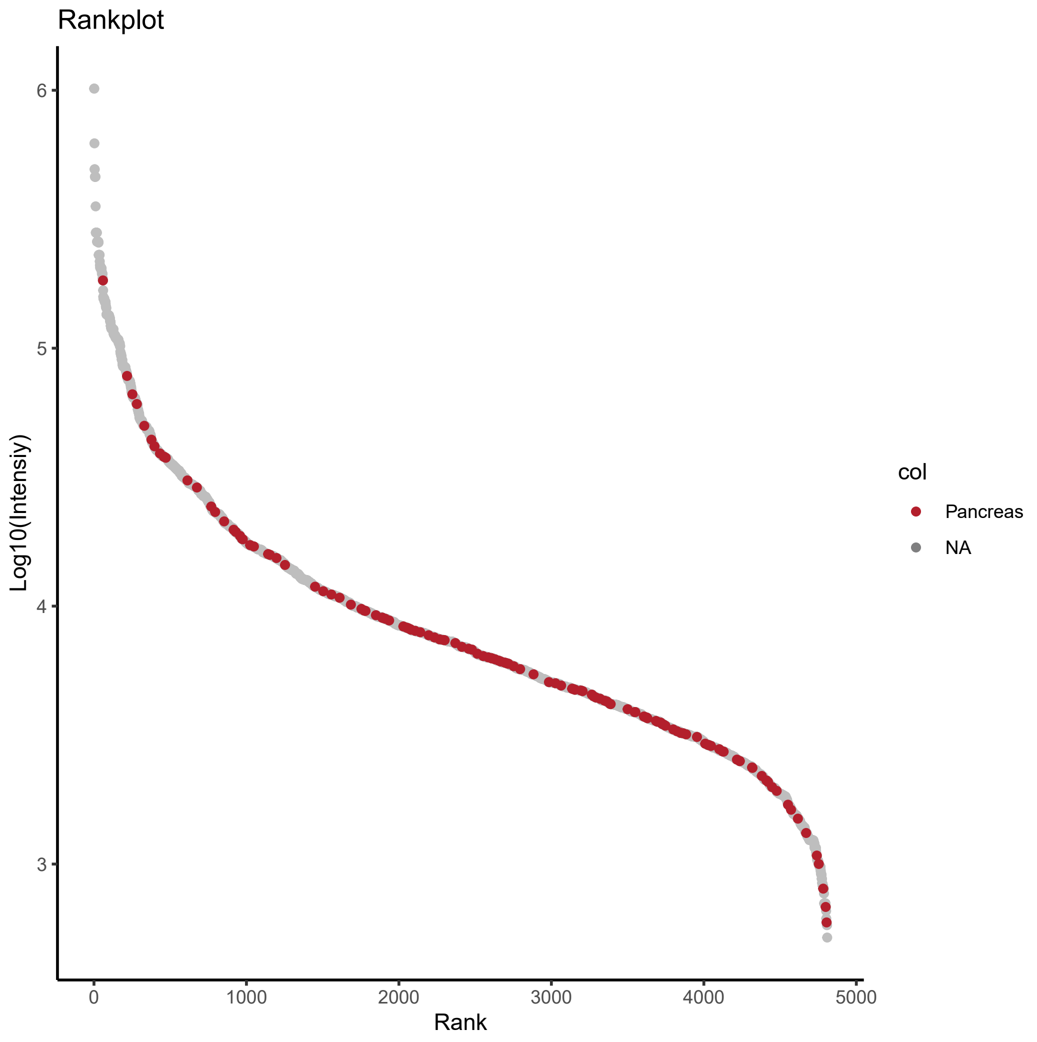
Rankplot
6
5
4
3
Log10(Intensiy)
0
1000
2000
3000
4000
5000
Rank
col
Pancreas
NA

U)

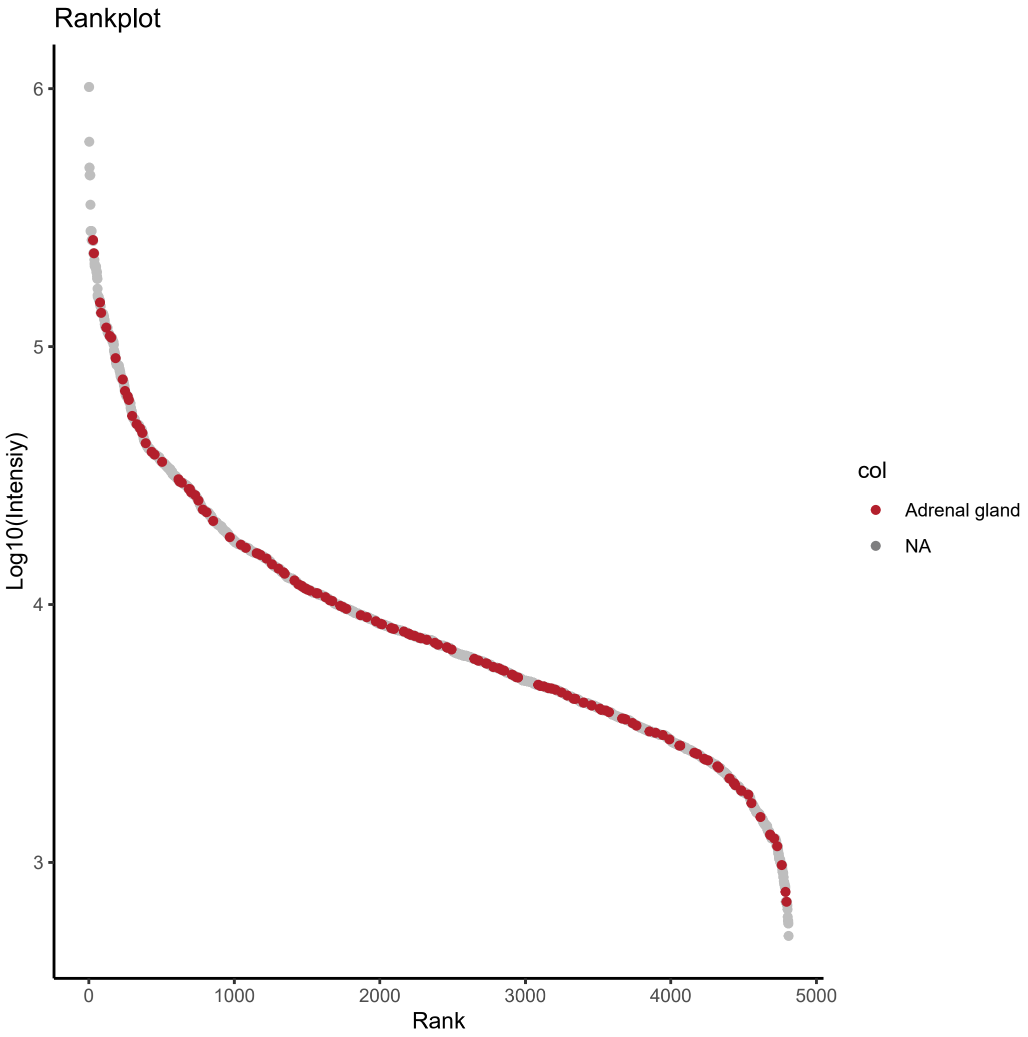
Rankplot
6
5
4
3
Log10(Intensiy)
0
1000
2000
3000
4000
5000
Rank
col
Adrenal gland
NA

V)

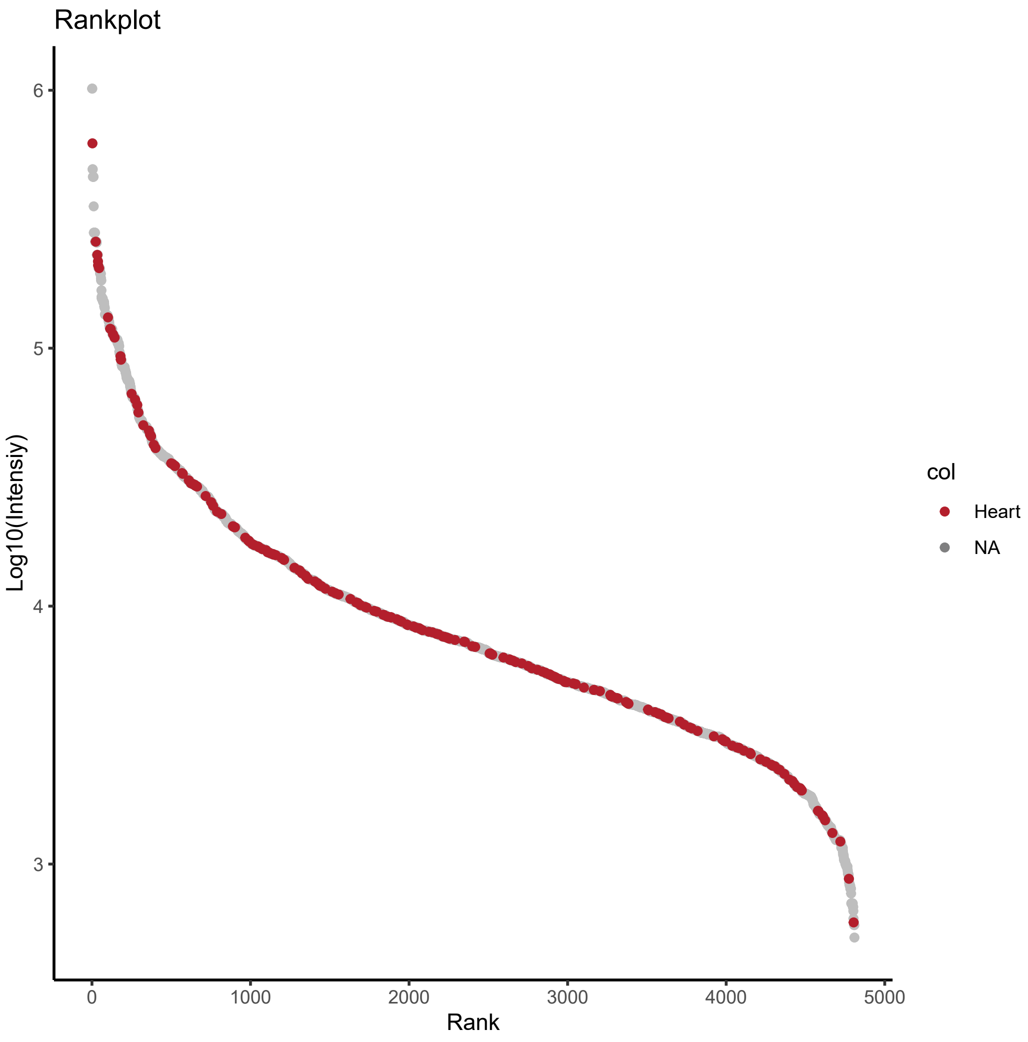
Rankplot
6
5
4
3
Log10(Intensiy)
0
1000
2000
3000
4000
5000
Rank
col
Heart
NA

W)

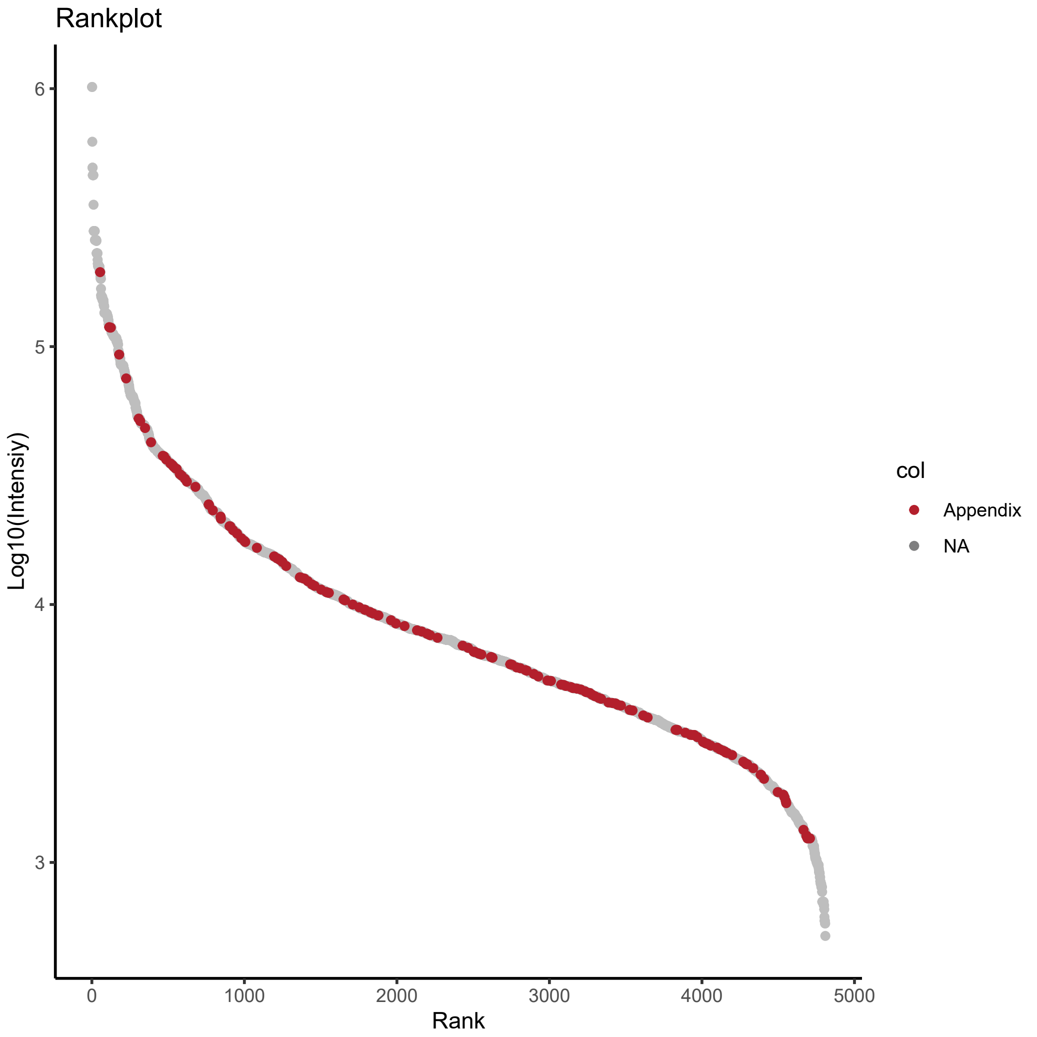
Rankplot
Log10(Intensiy)
6
5
4
3
0
1000
2000
3000
4000
5000
Rank
col
Appendix
NA

X)

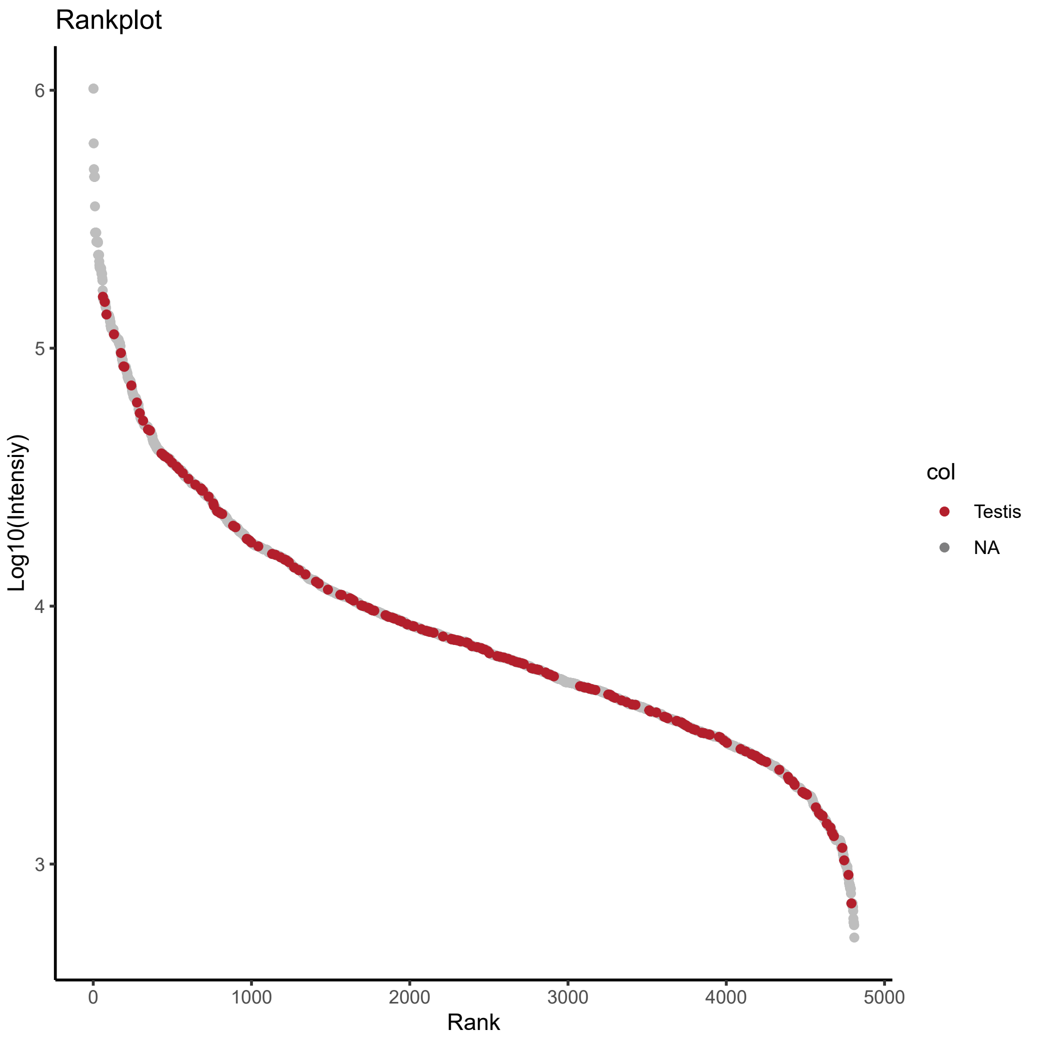
Rankplot
Log10(Intensiy)
6
5
4
3
0
1000
2000
3000
4000
5000
Rank
col
Testis
NA

Y)

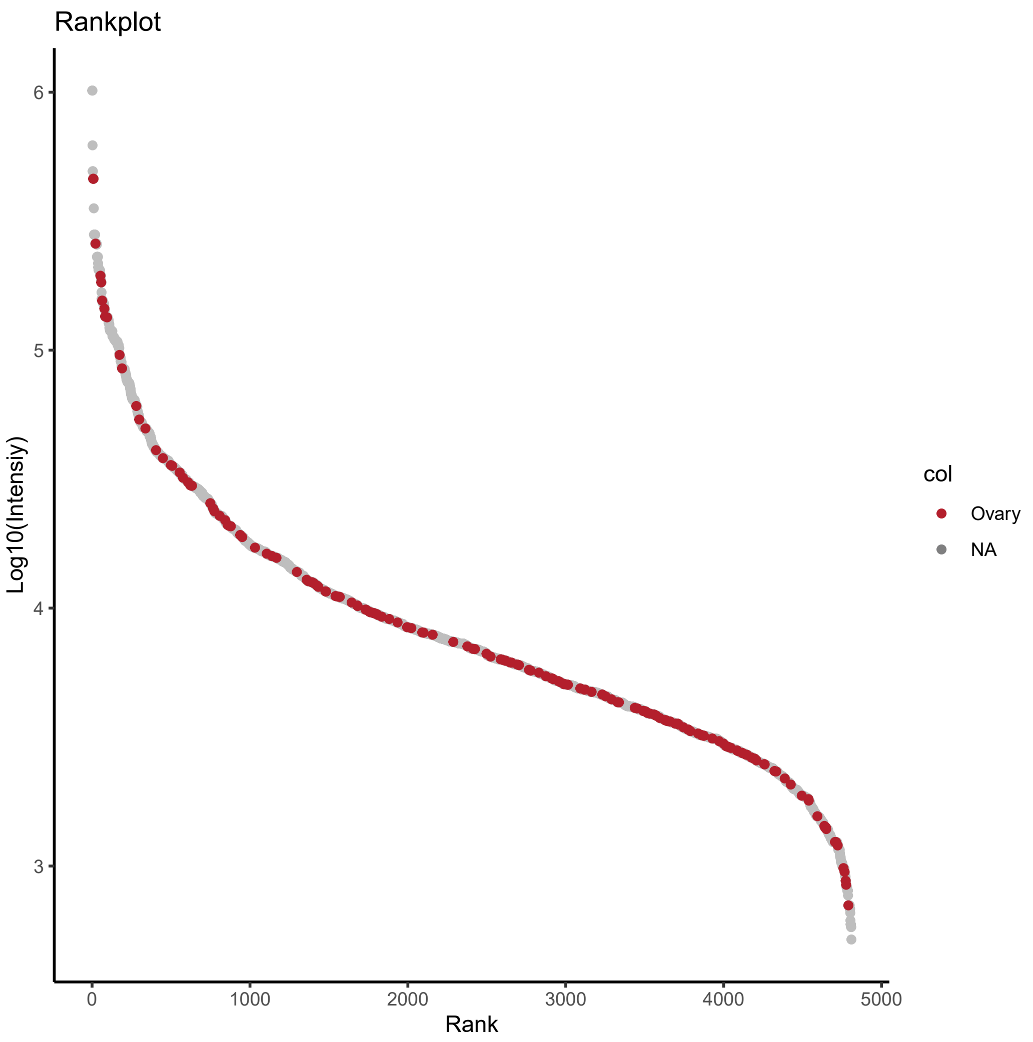

Rankplot
Log10(Intensiy)
6
5
4
3
0
1000
2000
3000
4000
5000
Rank
col
Ovary
NA

Z)

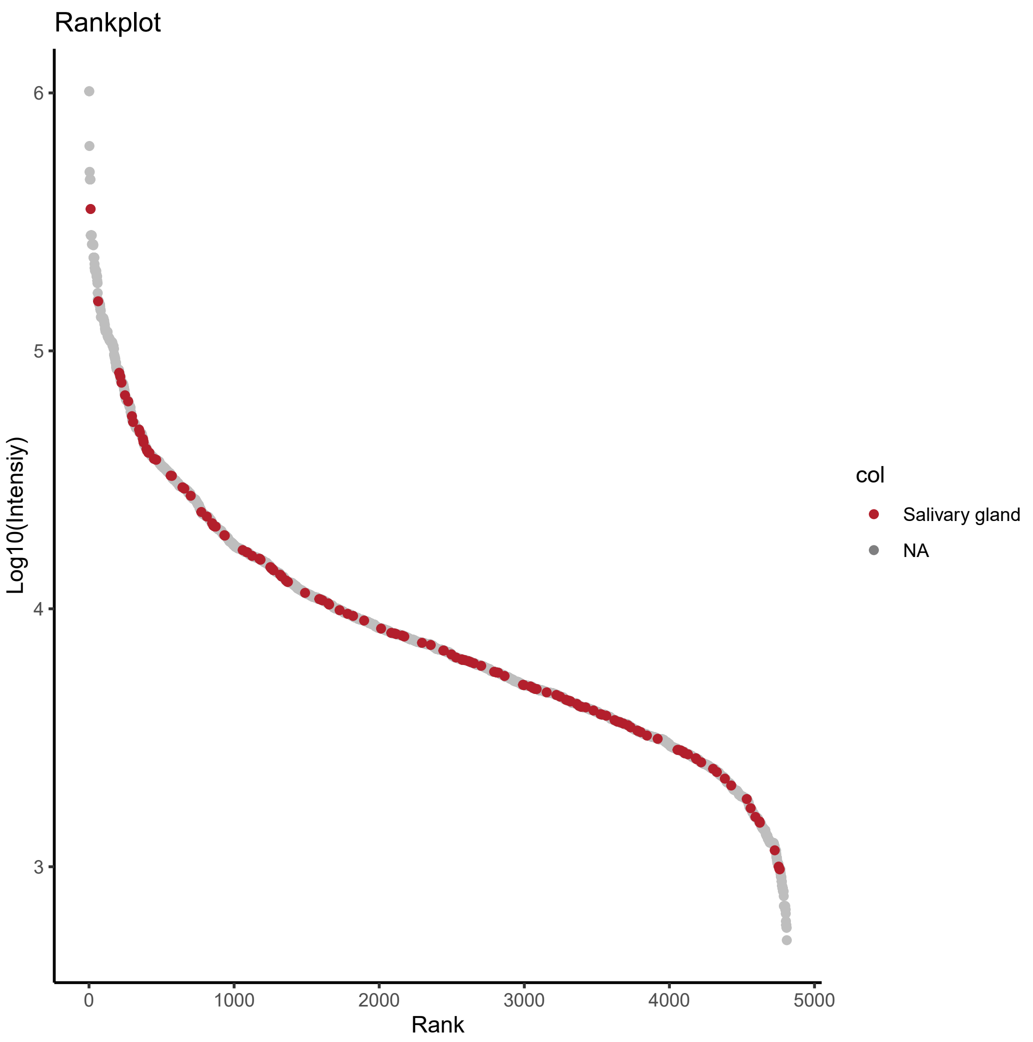

Rankplot
Log10(Intensiy)
6
5
4
3
0
1000
2000
3000
4000
5000
Rank
col
Salivary gland
NA

Supplementary Figure 3: (A)-(Z) Intensity rank plot of peptides derived from group enriched, tissue enhanced and tissue enriched genes colored by their respective organ assignment (dataset as in (Figure 4A)).
